# Personal

**Published:** 1896-04

**Authors:** 


### Personal.

DR. JOHN C. FISHER, late of Warsaw and physician-in-charge of the Warsaw Salt Baths and Sanitarium, has removed to California and, we understand, will assume relations with a sanatorium near San Francisco. Dr. Fisher is a qualified physician, ethical in his methods and every way competent to assume such place in the profession as he may choose. His career in the United States Marine Hospital service was an honorable one, and his resignation was accepted with regret by the supervising surgeon-general. We commend Dr. Fisher to the confidence of the medical profession of the Pacific coast.

---

DR. CHARLES A. L. REED, of Cincinnati, has been appointed by Governor Bushnell a member of the Ohio state board of medical registration and examination. Dr. Reed's appointment is an indication that the new board will be expected to do good work. It is difficult to see how it could have been possible for Governor Bushnell to have made a better appointment.

Dr. John Young Brown, Jr., formerly of Louisville and late first assistant physician in the Central Kentucky Asylum for the Insane, has removed to St. Louis. His office is at 507 North Spring avenue, and his hours are from 11 A. M. to 1 P. M. Dr. Brown will be certain to take a high position in the ranks of the medical profession of St. Louis.

---

Dr. Edward Clark, of Buffalo, has taken offices at the La Salle, 180 West Chippewa street, corner of Georgia street. Dr. Clark will continue to devote his attention to the treatment of diseases of the rectum. Hours: 11 A. M. to 2 P. M.

---

Dr. A. L. Benedict, of Buffalo, has been appointed associate editor of the *Philadelphia Medical and Surgical Reporter*. In additional to editorial work he will prepare the review of foreign progress in digestive diseases.

---

Dr. Frederick J. Mann, late of Watertown, N. Y., has been appointed assistant physician at the Hudson River State Hospital, Poughkeepsie, N. Y., and has entered upon the duties of his office.

---

Dr. Lillian C. Randall, of Buffalo, physician-in-charge of the Riverside Hospital, has removed her private office and residence from 41 Otis place to 502 Elmwood avenue.

---

Dr. Benjamin F. Griffith, of Springfield, Ill., has been elected recently president of the Illinois state board of health.

